# Eddy-Current-Induced Waveform Reconstruction by Metallic Probe Carriers in Magnetic Flux Leakage Inspection

**DOI:** 10.3390/s26134312

**Published:** 2026-07-07

**Authors:** Xiaoyuan Jiang, Bohan Jia, Yanhua Sun

**Affiliations:** School of Mechanical Science and Engineering, Huazhong University of Science and Technology, Wuhan 430074, China; jiangxy@hust.edu.cn (X.J.); d202280249@hust.edu.cn (B.J.)

**Keywords:** magnetic flux leakage (MFL), metallic probe carrier, motion-induced eddy current, waveform reconstruction, secondary magnetic field

## Abstract

**Highlights:**

- A metallic probe carrier is treated as an active near-field conductive boundary in MFL inspection.
- Motion-induced eddy currents generate a secondary magnetic field that reconstructs the measured waveform.
- Carrier material, scanning speed, and concave geometry jointly control peak shift, tailing, and distortion energy.
- Simulations and repeated-event experiments show waveform reconstruction rather than proportional attenuation.

**Abstract:**

Metallic probe carriers are commonly used in magnetic flux leakage (MFL) inspection to support sensing elements and maintain lift-off, but a conductive carrier located near the sensor can act as an active electromagnetic boundary. This study investigates the carrier-induced waveform reconstruction caused by such a conductive near-field boundary. A theoretical model is developed to describe the induced current, secondary magnetic field, and relaxation-related downstream memory generated when the carrier moves through a non-uniform leakage field. Transient finite-element simulations are used to examine the effects of carrier material, scanning speed, and concave carrier geometry. Compared with the air reference, aluminum and copper carriers produce stage-dependent waveform reconstruction, including valley modification, peak modulation, feature-position shift, and trailing-side extension. The quantitative waveform-deviation indicators increase with increasing speed and are further regulated by carrier geometry. Experimental results based on repeated magnetic response events confirm amplitude suppression, non-zero residual after amplitude matching, response broadening, and enhanced trailing asymmetry. These results demonstrate that the metallic probe carrier is not an electromagnetically transparent holder but an active near-field conductive boundary that should be considered in probe-carrier design and MFL signal interpretation.

## 1. Introduction

Ferromagnetic load-bearing components, including steel wire ropes, bridge cables, pipelines, tank floors, rail structures, and other steel members, commonly operate under coupled wear, corrosion, fatigue, impact, and environmental degradation. Once local material loss, cracking, or wire breakage exceed a tolerable threshold, the associated safety risk may increase disproportionately. Magnetic flux leakage (MFL) testing has therefore become a widely used electromagnetic nondestructive testing technique for ferromagnetic components because of its non-contact capability, high scanning efficiency, and engineering adaptability [1,2,3]. Recent studies have extended MFL inspection from defect detection toward quantitative imaging, nonlinear magnetization simulation, lift-off-tolerant sensing, and wire-rope damage evaluation. Representative work has addressed general MFL development and influencing factors [1], nonlinear MFL simulation under hysteresis and plastic deformation [2], wire-rope MFL imaging and quantitative evaluation [3], ferromagnetic lift-off regulation [4,5], sensor shielding [6], broken-wire identification [7], deeply buried defect detection [8], and temperature-dependent MFL modeling [9].

The measured MFL signal is not a direct copy of the defect source. It is shaped by local magnetization, defect geometry, boundary discontinuity, propagation path, lift-off attenuation, background-field coupling, sensor arrangement, structural interference, scanning speed, and nearby auxiliary structures. Recent studies have therefore focused on improving signal robustness, quantitative reconstruction, and multidirectional sensing. Shi et al. developed a dual-sensor strategy to resist lift-off disturbance in defect-depth evaluation [10], Zhang et al. proposed a defect-depth-field algorithm based on a discrete magnetic dipole model [11], and Liu et al. quantified the propagation characteristics of MFL signals for outer-surface defects in long-distance pipelines [12]. Sun et al. investigated broken-wire evaluation in bridge cables under lift-off uncertainty [13], while Shen et al. and Liu et al. developed composite sensing and magnetic focusing strategies for defect inspection [14,15]. Li et al. further introduced multidirectional MFL-based defect opening profile reconstruction [16].

These recent developments are built on earlier studies of MFL signal formation, field sensing, and defect characterization. Three-axis magnetic-field sensing was shown to improve defect characterization and was later verified through combined experimental and numerical analysis [17,18]. Finite-element calculation and three-dimensional simulation provided important tools for understanding MFL detector signals [19,20], while surface-breaking crack fields and local leakage-field capture were analyzed in early leakage-field studies [21,22]. Sensor-side observability was further improved through the magnetic-compression effect and the near-zero-background-field concept [23,24]. Subsequent studies examined metal-loss sizing and in-line simulation [25,26], weak MFL response and discontinuity-orientation effects [27,28], magnetic concentration and lift-off-tolerant testing [29,30], as well as surface-defect reconstruction, closely spaced pitting defects, multidirectional magneto-optical imaging, and complex-signal inversion [31,32,33,34]. These studies collectively indicate that MFL measurement is a reconstructed field response governed by source, propagation, sensing, and inspection conditions.

Over the last few years, MFL research has further moved toward profile reconstruction, intelligent diagnosis, high-speed compensation, and multimodal electromagnetic testing. Li et al. extended multidirectional MFL detection toward three-dimensional crack profile reconstruction [35], and Liang et al. proposed a homologous MFL and motion-induced eddy-current (MIEC) composite testing method in which MIEC generated in the inspected pipeline was intentionally used as a supplementary defect-sensitive signal for inner/outer defect discrimination and defect-angle-related identification [36]. Data-driven and physics-informed methods have also been introduced for anomaly detection and pipeline defect diagnosis [37,38]. Wang et al. investigated AC-MFL time-domain features for defect classification and quantification [39]. In wire-rope and cable inspection, Yang et al. carried out ILF diagnosis of steel wire ropes using physical information under a uniform circular array [40], Sun et al. designed a multilayer Hall-element sensor for bridge-cable broken-wire detection [41], and Ji et al. proposed a high-energy pulse-excitation MFL sensor for detecting internal defects in stay cables [42]. Meanwhile, high-speed MFL compensation, hybrid-network-based defect reconstruction, large-lift-off wire-rope inspection, and recent reviews on physical-model, AI, and multimodal fusion have further expanded the scope of MFL signal interpretation [43,44,45,46]. Classical eddy-current theory also provides the physical basis for understanding conductive-boundary effects near magnetic sources [47]. Thus, the scientific novelty of the present work does not lie in modifying classical induction or eddy-current theory but in locating this classical effect in a normally overlooked structural component of the MFL measurement chain.

Related dynamic effects have been discussed in high-speed MFL, motion-induced eddy-current testing, and dynamic magnetic coupling. These studies mainly address eddy currents generated in the inspected specimen, velocity-induced signal distortion, compensation strategies, or intentionally exploited MIEC signals, rather than eddy currents generated inside the probe carrier itself. Magnetic-permeability perturbation and eddy-current thermography also confirm that eddy-current-related effects can alter electromagnetic NDT signals in ferromagnetic components [48,49]. Dynamic magnetic coupling sensing further indicates that motion-related electromagnetic interaction can affect inspection signals under practical scanning conditions [50]. In contrast to existing research—such as Liang et al. [36], which deliberately employs specimen-generated MIEC as an inspection modality—the present work focuses on a fundamentally different object within the measurement chain: the unintended eddy currents generated inside the metallic probe carrier. This carrier is not part of the inspected specimen but rather an auxiliary structural component surrounding the sensing element. Therefore, the objective of the present study is not to introduce an additional defect-sensitive testing channel but to identify this often-overlooked component as an active near-field conductive boundary and to quantify how its induced secondary magnetic field influences MFL waveform formation and reconstruction. Furthermore, because this conductive carrier directly alters the near-field boundary conditions of the measurement region, its electromagnetic effects must be rigorously accounted for in probe-carrier design, waveform interpretation, and signal compensation.

To systematically address this issue, a mechanistic framework is proposed to evaluate carrier-induced waveform reconstruction during magnetic flux leakage (MFL) inspection. The contribution is therefore system level: it links carrier material, scanning speed, and concave geometry to measurable waveform-reconstruction indicators and to low-deviation probe-carrier design. The eddy-current-induced secondary-field effect is first formulated using a generalized induction model for a moving conductive carrier. An ideal steady magnetization state is then applied to isolate the motion-induced response generated as the sensor traverses a non-uniform leakage field. The model is subsequently expanded to incorporate dynamic magnetization via a source-evolution contribution. Finally, a diffusion-scanning criterion is introduced to correlate relaxation tailing, carrier geometry, and scan-speed dependence. The theoretical framework is corroborated by transient finite-element simulations and experimental validation, revealing that the metallic carrier substantially reshapes the MFL waveform—specifically altering its main peak, side lobes, zero-crossings, and downstream tail.

In this study, carrier-induced waveform reconstruction denotes the physical process in which the secondary field generated by carrier eddy currents superposes with the original leakage field and changes the measured waveform. The term wave-form-distortion indicator is reserved for quantitative deviation measures between the reconstructed waveform and the air reference waveform, such as normalized distortion energy, normalized waveform mismatch, or amplitude-matched residual.

**Figure 1:**
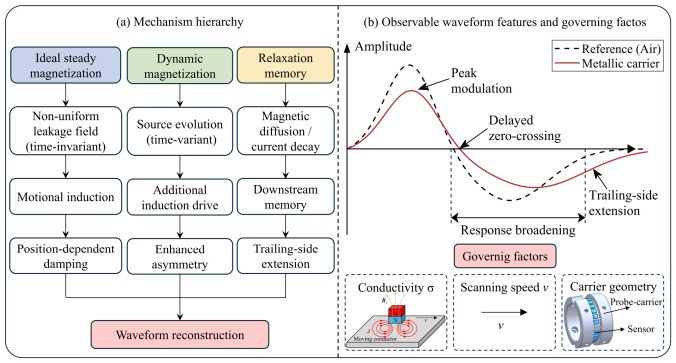
Mechanism analysis for waveform reconstruction. (**a**) Mechanism hierarchy of the primary physical processes driving the reconstruction. (**b**) Observable waveform features comparing a metallic carrier to an air reference, alongside key governing factors: conductivity, scanning speed, and carrier geometry.

The overall research logic and waveform-reconstruction pathway are summarized in Figure 1.

## 2. Theory

### 2.1. Carrier-Induced Eddy Currents and Secondary-Field Superposition

The conductive region of the metallic probe carrier is denoted by $\Omega_{c}$, with conductivity $\sigma_{c}$ and permeability $\mu_{c}$. The original leakage field without carrier-induced disturbance is $B_{s}$, and the carrier moves with velocity v relative to the specimen. The corresponding magnetic vector potential is $A_{s}$, with $B_{s}=\nabla \times A_{s}$.

The following assumptions are adopted in this reduced induction model. First, the characteristic electromagnetic wavelength is much larger than the probe-carrier dimension, so the displacement-current and radiation terms are neglected, and the field interaction is treated in the magneto-quasi-static regime. Second, the carrier is regarded as a finite, rigid, isotropic conductor moving with a prescribed velocity v. Third, $B_{s}$ is assumed to be spatially differentiable within $\Omega_{c}$ and represents the source leakage field before the secondary field of the carrier is superposed. Fourth, the aluminum and copper carriers considered in this study are non-ferromagnetic conductors with $\mu_{c}\approx \mu_{0}$; therefore, the permeability contrast of the carrier is not treated as an additional magnetic-concentration mechanism in the reduced model. The nonlinear permeability of the steel specimen and yokes is included in the source-field solution used to obtain $B_{s}$. Finally, the carrier-induced secondary field is assumed not to significantly alter the magnetization state of the specimen and yokes, so that weak-feedback superposition is applicable at the sensing point.

(1) $$J_{c}=\sigma_{c}\left(-\nabla \varphi_{c}-\frac{\partial A_{s}}{\partial t}+v\times B_{s}\right),\;\;B_{s}=\nabla \times A_{s}$$

Here, $-\nabla \varphi_{c}-\partial A_{s}/\partial t$ is the electric-field contribution associated with the scalar and vector potentials, while $v\times B_{s}$ is the motional induction term caused by carrier motion through the leakage field. The scalar potential $\varphi_{c}$ is not an externally applied voltage. It represents charge redistribution in the finite conductor and provides the potential correction required for current closure.

Because the carrier is electrically isolated and no external circuit is connected to it, the induced current must satisfy charge conservation inside $\Omega_{c}$ and a no-through-current condition on the carrier boundary. Thus,

(2) $$\nabla \cdot J_{c}=0\;in\Omega_{c},\;\;J_{c}\cdot n=0\;on\;\partial \Omega_{c}.$$

where **n** is the outward unit normal vector on $\partial \Omega_{c}$. Equation (2) determines the scalar-potential distribution required to close the induced eddy-current loops within the finite carrier.

Once $J_{c}$ is obtained, the carrier-induced secondary magnetic flux density at the sensing position $r_{s}$ is evaluated using the magneto-quasi-static Biot–Savart relation,

(3) $$B_{sec}\left(r_{s},t\right)=\frac{\mu_{0}}{4\pi }\int_{\Omega_{c}}\frac{J_{c}\left(r^{\prime },t\right)\times \left(r_{s}-r^{\prime }\right)}{{\left|r_{s}-r^{\prime }\right|}^{3}}d\Omega^{\prime }.$$

The use of $\mu_{0}$ in Equation (3) is consistent with the non-ferromagnetic carrier materials investigated in this work. If a ferromagnetic carrier with a significant permeability contrast were used, the carrier magnetization would have to be included explicitly by solving the full magnetic-field problem rather than using the reduced secondary-field expression in Equation (3).

The magnetic flux density measured by the sensing element is then expressed as the superposition of the original leakage field and the carrier-induced secondary field,

(4) $$B_{m}\left(r_{s},t\right)=B_{s}\left(r_{s},t\right)+B_{sec}\left(r_{s},t\right).$$

Therefore, Equation (1) follows from moving-conductor Ohm’s law, Equation (2) follows from charge conservation and electrical insulation of the finite carrier, Equation (3) follows from the magneto-quasi-static secondary field generated by the induced current, and Equation (4) follows from the weak-feedback superposition approximation. These equations indicate that the metallic carrier is not an electromagnetically transparent holder. When $\sigma_{c}$ approaches zero, $J_{c}$ and $B_{sec}$ become negligible. When $\sigma_{c}$ is high, the induced current is strengthened, and the resulting secondary magnetic field can reconstruct the measured MFL waveform.

**Figure 2:**
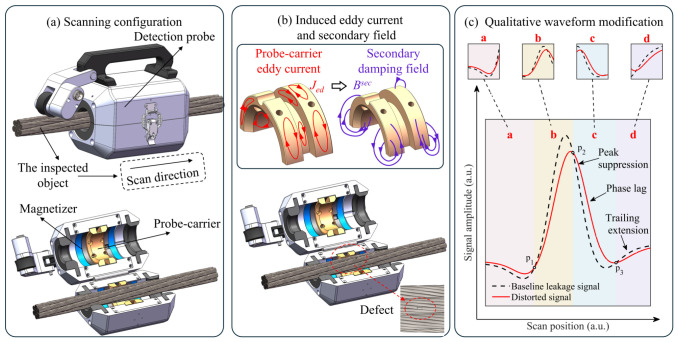
Physical origin of carrier-induced waveform reconstruction by the metallic probe carrier. (**a**) Schematic scanning configuration of the defect, metallic probe carrier, and embedded magnetic sensing element. (**b**) Induced eddy-current loops and the associated secondary magnetic field inside the conductive carrier. (**c**) Qualitative comparison between the baseline leakage signal and the reconstructed signal measured under the metallic probe carrier.

The near-field eddy-current interaction induced by the metallic probe carrier is illustrated in Figure 2.

### 2.2. Response Under Ideal Steady Magnetization

In the ideal steady-magnetization case, the defect-equivalent source, magnetization state, and excitation field remain unchanged during carrier motion. In the specimen-fixed frame, the source field is spatially non-uniform but time-invariant,

(5) $$\frac{\partial A_{s}}{\partial t}=0,\;\;\frac{\partial B_{s}}{\partial t}=0.$$

The carrier, however, moves through this non-uniform leakage field and therefore samples different field values along the scanning path. Under the assumptions stated in Section 2.1, Equation (1) reduces to

(6) $$J_{c0}=\sigma_{c}\left(-\nabla \varphi_{c0}+v\times B_{s}\right).$$

Here, the subscript 0 denotes the carrier current induced by the steady source field. The scalar potential $\varphi_{c0}$ adjusts to satisfy current closure in the finite carrier. Therefore, a spatially uniform motional electric field mainly produces charge redistribution and is largely cancelled by $-\nabla \varphi_{c0}$. The sustained eddy-current loop is driven by the non-conservative part of the motional term $v\times B_{s}$, which becomes non-zero when the carrier moves through a spatially varying leakage field.

The corresponding secondary and measured fields are

(7) $$B_{sec,0}\left(r_{s},t\right)=\frac{\mu_{0}}{4\pi }\int_{\Omega_{c}}\frac{J_{c0}\left(r^{\prime },t\right)\times \left(r_{s}-r^{\prime }\right)}{{\left|r_{s}-r^{\prime }\right|}^{3}}\;d\Omega^{\prime }.$$

(8) $$B_{m,0}\left(r_{s},t\right)=B_{s}\left(r_{s},t\right)+B_{sec,0}\left(r_{s},t\right).$$

The order-of-magnitude estimate of $J_{c0}$ can be obtained from the circulation of the motional electric field around a representative closed current path ***C*** in the carrier. Let

(9) $$E_{m}=v\times B_{s}.$$

For rigid motion with spatially uniform v, and using $\nabla \cdot B_{s}=0$, the curl of the motional term is

(10) $$\nabla \times E_{m}=\nabla \times \left(v\times B_{s}\right)=-\left(v\cdot \nabla \right)B_{s}.$$

Thus, in the order-of-magnitude sense,

(11) $$\left|\nabla \times E_{m}\right|∼v\left|\nabla B_{s}\right|,$$

where $\left|\nabla B_{s}\right|$ denotes the characteristic magnitude of the spatial gradient of the source leakage field sampled by the current loop.

According to Stokes’ theorem, the motional electromotive force around a loop with characteristic length $L_{e}$ is

(12) $$E_{m}=\oint_{C}E_{m}\cdot dl=\iint_{S_{C}}\left(\nabla \times E_{m}\right)\cdot n_{S}dS∼v\left|\nabla B_{s}\right|L_{e}^{2},$$

where $S_{C}$ is the surface enclosed by C, and $n_{S}$ is the unit normal vector of $S_{C}$. The loop-averaged non-conservative driving electric field is therefore

(13) $$E_{eff}∼\frac{E_{m}}{L_{e}}∼vL_{e}\left|\nabla B_{s}\right|.$$

Substitution into Ohm’s law gives the order estimate

(14) $$\left|J_{c0}\right|∼\sigma_{c}vL_{e}\left|\nabla B_{s}\right|.$$

Here, $L_{e}$ is the effective length of the dominant eddy-current loop that is electromagnetically coupled to the sensing point. Operationally, it can be interpreted as the scan-direction projected distance between two portions of the current loop that sample different values of the leakage field. For the concave carrier considered in this work, $L_{e}$ is of the order of the conductive path surrounding Point A and is controlled mainly by the bottom-width and side-wall geometry, rather than by the full external perimeter of the carrier.

Equation (14) shows that a spatially uniform leakage field does not generate a sustained closed eddy-current response after scalar-potential redistribution, whereas a spatially varying leakage field produces a finite-loop electromotive force. Therefore, the carrier response is expected to be strongest in waveform regions where the leakage-field gradient sampled by the carrier is large, such as side-lobe transitions, main-response slopes, and zero-crossing neighborhoods. This explains why the conductive carrier reconstructs the measured waveform in a position-dependent manner instead of producing a uniform amplitude scaling.

The estimate in Equation (14) is valid when the source leakage field can be locally approximated by a first-order spatial expansion over the effective loop length $L_{e}$, the carrier velocity can be regarded as locally constant, the magnetic skin depth is not much smaller than the characteristic conducting thickness of the carrier, and the induced secondary field remains a perturbation to the source leakage field. If these conditions are violated, $L_{e}$ and the effective conducting cross-section should be replaced by geometry- and frequency-dependent effective quantities obtained from the full electromagnetic solution.

**Figure 3:**
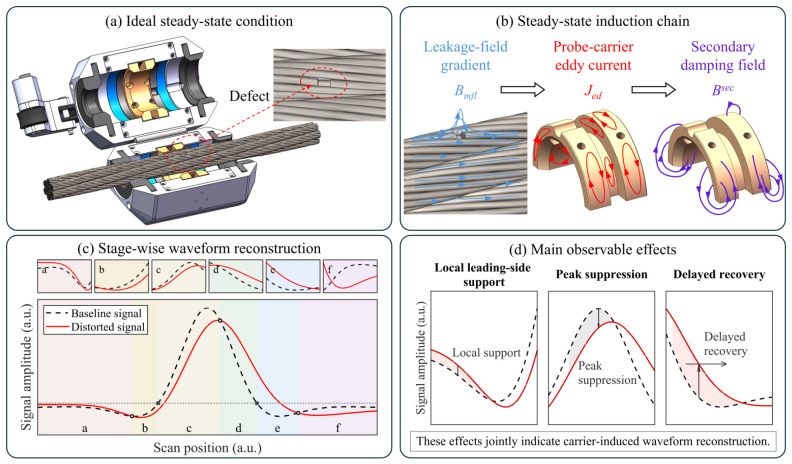
Response mechanism and staged waveform reconstruction under ideal steady magnetization. (**a**) Ideal steady-state condition with a spatially non-uniform but time-invariant source field. (**b**) Relationship among field-gradient-driven induction, carrier eddy current, and secondary field. (**c**) Stage-wise waveform evolution under steady scanning. (**d**) Summary of local side-lobe compensation, main-peak suppression, and delayed recovery in the trailing region.

The response mechanism under ideal steady magnetization is further summarized in Figure 3.

### 2.3. Additional Waveform Reconstruction Under Dynamic Magnetization

In practical inspection, the magnetizer, carrier, and defect region may move relative to one another. The local magnetization state near the defect can then evolve with scan position, and the source field becomes spatiotemporal,

(15) $$B_{s}=B_{s}\left(r,t\right),\;\;\frac{\partial A_{s}}{\partial t}\neq 0.$$

The induced current can be decomposed as

(16) $$J_{c}=J_{c0}+J_{cd}.$$

(17) $$J_{cd}=\sigma_{c}\left(-\nabla \varphi_{cd}-\frac{\partial A_{s}}{\partial t}\right).$$

The dynamic increment of the secondary field is

(18) $$\Delta B_{sec,d}\left(r_{s},t\right)=\frac{\mu_{0}}{4\pi }\int_{\Omega_{c}}\frac{J_{cd}\left(r^{\prime },t\right)\times \left(r_{s}-r^{\prime }\right)}{{\left|r_{s}-r^{\prime }\right|}^{3}}\;d\Omega^{\prime }$$

and the measured field becomes

(19) $$B_{m,d}=B_{s}+B_{sec,0}+\Delta B_{sec,d}.$$

The dynamic term does not replace the steady motional mechanism; it redistributes the driving strength of the carrier response. During defect approach and departure, the source-evolution contribution can differ between the leading and trailing sides, strengthening waveform asymmetry and downstream tailing.

**Figure 4:**
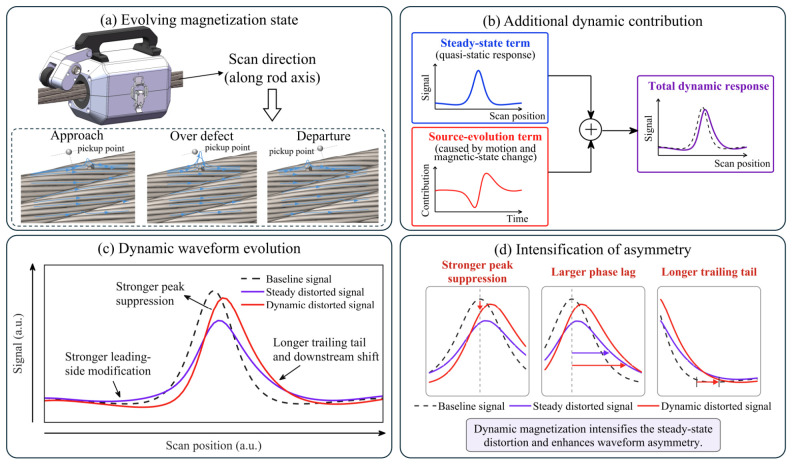
Dynamic magnetization and enhancement of asymmetric waveform reconstruction. (**a**) Evolving magnetization state during defect approach and departure. (**b**) Comparison between the steady motional contribution and the additional source-evolution contribution. (**c**) Stage-wise waveform evolution under dynamic magnetization. (**d**) Direct comparison between steady and dynamic reconstructed waveforms, showing deeper peak compression, stronger trailing-side support, and longer tailing in the dynamic case.

The additional waveform reconstruction associated with dynamic magnetization is shown in Figure 4.

### 2.4. Relaxation Tailing, Geometric Influence, and Waveform-Reconstruction Criterion

The induced current in a real conductor requires finite time to diffuse, redistribute, and decay. However, this temporal behavior should not be interpreted as a single universal material constant under all measurement conditions. In the present framework, we therefore distinguish between a material-diffusion lower-bound timescale and an effective waveform-memory timescale of the complete measurement chain.

For a carrier with characteristic conducting thickness *l_c_*, a one-dimensional through-thickness magnetic diffusion timescale can be estimated as

(20) $$\tau_{d,est}\approx \frac{\mu_{c}\sigma_{c}l_{c}^{2}}{\pi^{2}}.$$

This estimate represents a lower-bound diffusion time associated with simplified through-thickness magnetic diffusion. It does not include contributions from three-dimensional current-loop closure, distributed current paths, sensor-carrier coupling distance, or signal processing effects and therefore provides only a baseline physical reference.

A first-order relaxation representation of the secondary field is

(21) $$\tau \frac{dB_{sec}}{dt}+B_{sec}=K_{c}D\left(t\right)$$

where $K_{c}$ is a material-geometry coupling operator, and **D**(*t*) denotes the effective induction drive containing both motional and source-evolution contributions. Carrier geometry controls the current-loop path, conducting cross-section, loop resistance, current centroid, and coupling distance to the sensor. A compact coupling measure is

(22) $$K_{c}\left(r_{s}\right)\propto \int_{\Omega_{c}}\sigma_{c}G_{B}\left(r_{s},r^{\prime }\right)\;L_{e}\left(r^{\prime }\right)\;d\Omega^{\prime }$$

where *G_B_* denotes the magnetic-field propagation kernel from the current path to the sensing point. The competition between carrier relaxation and scanning time can be described by

(23) $$\Lambda \left(\tau \right)=\frac{v\tau }{W_{d}}.$$

where *v* is the scanning velocity, *W_d_* is the characteristic defect-response width, and τ is explicitly defined as the selected memory timescale for each specific analysis context, i.e., either the material-diffusion estimate $\tau_{d,est}$ or the effective waveform-memory time $\tau_{fit}$.

Two distinct interpretations of τ are explicitly distinguished in this work: When $\tau =\tau_{d,est}$, Equation (23) defines a material-diffusion lower-bound ratio $\Lambda_{d,est}=v\tau_{d,est}/W_{d}$. When $\tau =\tau_{eff}$, representing the effective waveform-memory of the complete measurement chain, the ratio becomes $\Lambda_{eff}=v\tau_{eff}/W_{d}$.

In the present study, the experimentally fitted relaxation parameter $\tau_{fit}$, introduced in Section 4.4, is interpreted as an estimate of $\tau_{eff}$ rather than a material constant of the conductive carrier.

When evaluated using a generic timescale τ, the criterion provides a transition description rather than a strict material law. Specifically, when *Λ_d_* ≪ 1, the memory associated with the selected timescale decays within a small fraction of the defect-response width, and relaxation-controlled downstream tailing is weak. However, localized waveform modulation may still occur due to instantaneous secondary-field superposition in regions of high magnetic-field gradient. When *Λ_d_* ≳ 1, the selected memory timescale becomes comparable to the scanning time across the defect-response region, leading to peak suppression, feature displacement, and pronounced trailing-side extension. The corresponding critical speed is

(24) $$v_{cr}\approx \frac{W_{d}}{\tau }.$$

It should be emphasized that Equation (24) is not associated with a single fixed material constant but depends on the selected interpretation of τ, i.e., either the material-diffusion lower bound $\tau_{d,est}$ or the effective waveform-memory time $\tau_{eff}$.

Equations (20)–(24) establish three testable consequences: the conductive carrier should cause waveform reshaping rather than proportional attenuation; the residual after amplitude matching should remain non-zero in high-gradient and downstream regions; and relaxation should broaden the response and increase trailing asymmetry.

**Figure 5:**
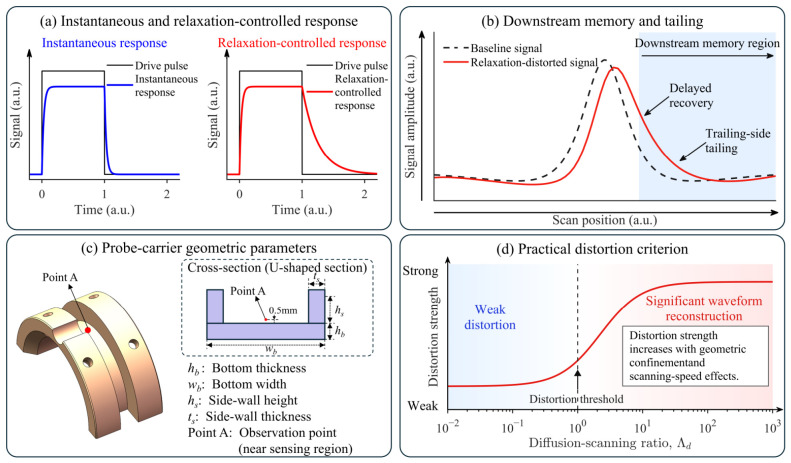
Diffusion-scanning ratio Λ(τ) and its relationship to measurable waveform indicators (peak shift, distortion energy, trailing asymmetry). (**a**) Comparison between an instantaneous response and a relaxation-controlled response. (**b**) Downstream memory and trailing-side signal extension. (**c**) Definition of the main geometric parameters of the metallic probe carrier and sensing point. (**d**) Regime map of Λ(τ) illustrating the transition from weak to significant waveform reconstruction.

The relaxation-controlled tailing mechanism, carrier-geometrical influence, and diffusion-scanning criterion are summarized in Figure 5.

## 3. Finite-Element Corroboration

### 3.1. Numerical Model and Simulation Scheme

A two-dimensional axisymmetric transient finite-element model was established to investigate carrier-induced electromagnetic distortion. The model consisted of a 1020-steel rod, a localized surface defect, an N52 permanent magnet, 1020-steel yokes, and a concave probe carrier located in the near-field sensing region. The rod was treated as the rotationally symmetric ferromagnetic specimen. The local surface defect generated a non-uniform leakage field during scanning. Point A was defined in the central recess of the carrier and used as the magnetic-field extraction point corresponding to the sensing element. In this axisymmetric representation, the concave carrier cross-section is revolved about the specimen axis and therefore represents a circumferentially continuous ring-like conductive carrier rather than an open three-dimensional probe holder with finite axial ends or interrupted conductive paths.

The carrier geometry was described by bottom thickness *h_b_*, bottom width *w_b_*, side-wall height *h*_s_, and side-wall thickness *t_s_*. These dimensions control the conducting cross-section, current-loop path, resistance, and coupling distance between the induced current and Point A. Three carrier materials were compared: air, aluminum, and copper. Air served as the non-conductive reference, while aluminum and copper represented conductive carriers with different induced-current strengths. Scanning velocity was varied to evaluate dynamic enhancement. The nonlinear magnetic properties of 1020 steel were introduced using the measured B-H curve and the corresponding μ-H relation.

During transient simulation, the magnetizer and carrier moved along the axial direction of the steel rod. The extracted Bz waveform at Point A was converted into a defect-related response by subtracting the pre-defect baseline,

(25) $$\Delta B_{z}\left(t\right)=B_{z}\left(t\right)-{\bar{B}}_{z,bg}.$$

For comparison among velocities, the time coordinate was converted into equivalent scanning position,

(26) $$x=v\left(t-t_{0}\right).$$

**Figure 6:**
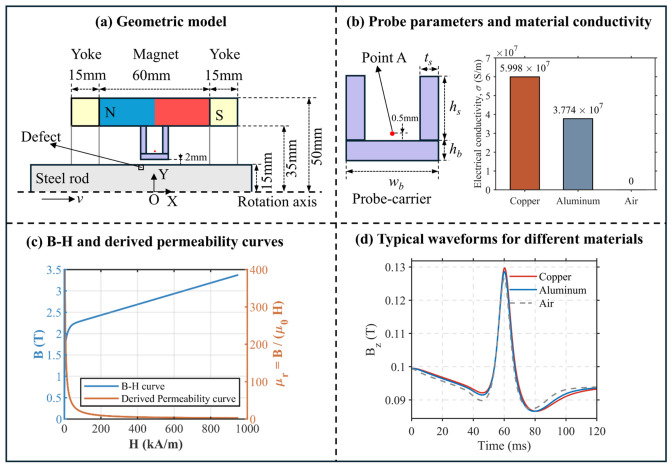
Finite-element simulation scheme and data extraction route. (**a**) Geometrical configuration and parameter setting of the two-dimensional axisymmetric transient model, including the steel rod, defect, magnetizer, yokes, probe carrier, rotation axis, motion direction, and main dimensions. (**b**) Definition of the probe-carrier variables, including the bottom thickness *h_b_*, bottom width *w_b_*, side-wall height *h_s_*, side-wall thickness *t_s_*, and the extraction point Point A. (**c**) Nonlinear magnetic material properties of 1020 steel, including the *B-H* curve and the corresponding *μ-H* curve used for the steel rod and yokes. (**d**) Data extraction and waveform processing route, including the extraction of *Bz* at Point A, baseline subtraction to obtain Δ*Bz*, conversion from time to scanning position, and representative waveform indicators used for the following analysis.

The finite-element configuration and data-extraction route are given in Figure 6, and the main simulation parameters and variables are summarized in Table 1.

### 3.2. Conductivity-Dependent Waveform Reconstruction and Side-Wall-Thickness Effect

The conductivity-dependent response was examined under a representative scanning speed and carrier geometry. Air was used as the non-conductive reference, whereas aluminum and copper were used as conductive carriers. The extracted Δ*B_z_* waveforms were divided into characteristic regions, including the negative-response region, rising transition, main-response region, peak neighborhood, post-peak recovery, and trailing region.

The conductive carriers do not modify the reference waveform by a fixed proportional factor. In the early negative-response region, aluminum and copper reduce the negative excursion relative to air. In the rising region, the conductive curves gradually deviate from the non-conductive response, indicating that the secondary field changes the local growth process. Around the main peak, both magnitude and position are modified. After the peak, the conductive cases maintain a different recovery trajectory and show a more extended downstream response. These features confirm stage-dependent waveform reconstruction rather than uniform amplitude attenuation.

**Figure 7:**
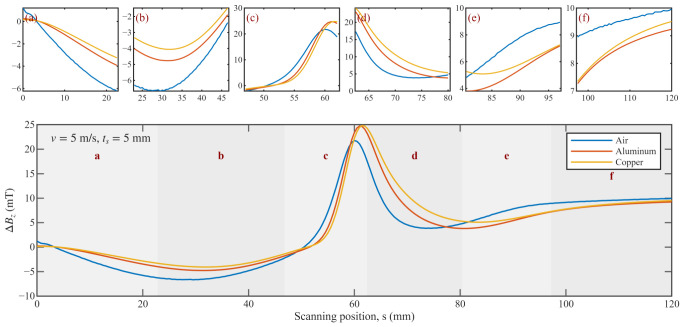
Conductivity-dependent staged waveform reconstruction under the representative baseline condition. Full Δ*B_z_* waveforms of air, aluminum, and copper carriers are shown together with six local enlarged windows. The red letters a–f in the main waveform mark the staged response regions, and the inset labels (a)–(f) denote the corresponding local enlarged windows: (a) early negative-response region; (b) negative-valley region; (c) rising-transition region; (d) main-response and peak-neighborhood region; (e) post-peak recovery region; and (f) downstream trailing region. The air case represents the non-conductive reference, whereas aluminum and copper represent conductive probe carriers with different eddy-current responses. The conductive carriers modify the negative valley, rising segment, main-response region, peak position, and recovery tail in different ways, indicating stage-dependent waveform reconstruction rather than uniform amplitude attenuation.

The conductivity-dependent staged waveform reconstruction is compared in Figure 7.

A direct comparison between the reduced theoretical calculation and the finite-element response was used as a mechanism-level corroboration. The reduced calculation describes the idealized induction response of a moving conductive boundary in a non-uniform leakage field, whereas the finite-element model includes the actual axisymmetric geometry, finite-domain current closure, nonlinear material properties, boundary conditions, and sensor-carrier coupling distance. At the feature level, both descriptions show that the copper response differs from the air response in the high-gradient region, near the main response, and during downstream recovery. This consistency supports the secondary-field origin of the reconstructed waveform and indicates that the conductive carrier modifies the measured response in a stage-dependent manner rather than by uniform amplitude scaling.

**Figure 8:**
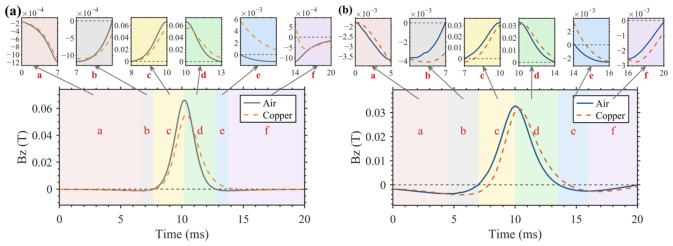
Correspondence between reduced theoretical calculation and finite-element simulation for stage-dependent waveform reconstruction. (**a**) Reduced theoretical calculation under air and copper conditions, showing local waveform modification in six characteristic stages. (**b**) Finite-element simulation under air and copper conditions, showing consistent stage-dependent reconstruction of the measured Δ*B_z_* response. In both panels, the red letters a–f denote the six characteristic staged waveform regions: a, early negative response; b, negative valley; c, rising transition; d, main-response and peak-neighborhood region; e, post-peak recovery; and f, downstream trailing response. The enlarged views indicate that the conductive carrier modifies the high-gradient region, main-response region, and downstream recovery region rather than producing uniform amplitude scaling.

Figure 8 further shows the correspondence between the reduced theoretical calculation and the finite-element result.

To make this comparison quantitative, the copper–air difference waveforms from Figure 8a,b were extracted from the source curves and interpolated onto a common time grid. Let $B_{Air}^{red}$ and $B_{Cu}^{red}$ denote the reduced-calculation waveforms, and let $B_{Air}^{FEM}$ and $B_{Cu}^{FEM}$ denote the finite-element waveforms. The conductive-carrier contribution was defined as

(27) $$\Delta B_{red}\left(t_{i}\right)=B_{Cu}^{red}\left(t_{i}\right)-B_{Air}^{red}\left(t_{i}\right),\;\;\Delta B_{FEM}\left(t_{i}\right)=B_{Cu}^{FEM}\left(t_{i}\right)-B_{Air}^{FEM}\left(t_{i}\right).$$

Each difference waveform was normalized to zero mean and unit peak-to-peak amplitude as

(28) $${\hat{\Delta B}}_{k,i}=\frac{\Delta B_{k}\left(t_{i}\right)-\bar{\Delta B_{k}}}{\underset{i}{max}\Delta B_{k}\left(t_{i}\right)-\underset{i}{min}\Delta B_{k}\left(t_{i}\right)},\;\;k\in \{red,FEM\}.$$

The waveform-level agreement was quantified by the normalized correlation coefficient and normalized mismatch,

(29) $$\rho_{\Delta }=\frac{\sum_{i}{\hat{\Delta B}}_{red,i}{\hat{\Delta B}}_{FEM,i}}{{\left(\sum_{i}{\hat{\Delta B}}_{red,i}^{2}\right)}^{1/2}{\left(\sum_{i}{\hat{\Delta B}}_{FEM,i}^{2}\right)}^{1/2}},\;\;E_{mis}=\frac{{∥{\hat{\Delta B}}_{red}-{\hat{\Delta B}}_{FEM}∥}_{2}}{{∥{\hat{\Delta B}}_{FEM}∥}_{2}}.$$

The air-to-copper peak delay and the peak-shift error were defined as

(30) $$t_{p,m}^{k}=arg\underset{t}{max}B_{m}^{k}\left(t\right),\;\;\Delta t_{p,k}=t_{p,Cu}^{k}-t_{p,Air}^{k},\;\;e_{p}=\left|\Delta t_{p,red}-\Delta t_{p,FEM}\right|.$$

where m∈Air,Cu and k∈red,FEM. Using these definitions, the normalized waveform correlation coefficient was $\rho_{\Delta }=0.70$, and the normalized waveform mismatch was $E_{mis}=0.71$. In addition, the air-to-copper peak delay was 0.16 ms in the reduced calculation and 0.25 ms in the finite-element result, giving a peak-shift error of $e_{p}=0.09$ ms. These results indicate moderate waveform-level agreement and confirm that the reduced calculation captures the main stage-dependent reconstruction trend. The remaining mismatch is expected because the reduced calculation omits exact finite-element boundary conditions, detailed finite-domain current closure, nonlinear material coupling, and the full sensor-carrier coupling geometry. Thus, Figure 8 supports mechanism-level corroboration, but it should not be interpreted as a calibrated analytical prediction of the full finite-element amplitude.

The influence of side-wall thickness *t_s_* was then evaluated. For the air reference, positive peak, negative-valley magnitude, and peak position remain almost unchanged as *t_s_* varies, indicating that geometry alone has limited effect when the carrier is non-conductive. Aluminum and copper show clear *t_s_* -dependent variation. The negative-valley magnitude and normalized distortion energy exhibit non-monotonic tendencies, indicating that a larger conducting region can enhance the induced current while also redistributing the current centroid, increasing loop resistance, or reducing near-field coupling to Point A.

**Figure 9:**
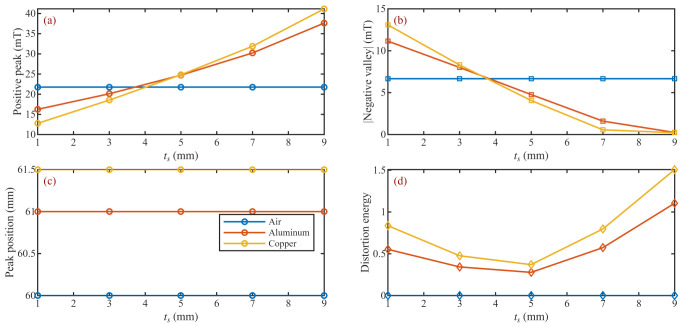
Quantitative influence of side-wall thickness *t_s_* on material-dependent waveform characteristics. (**a**) Positive peak. (**b**) Negative-valley magnitude. (**c**) Peak position. (**d**) Normalized distortion energy. The air reference remains nearly unchanged with *t_s_*, whereas aluminum and copper show significant *t_s_*-dependent variations in peak amplitude, valley magnitude, feature position, and waveform distortion energy.

The influence of side-wall thickness on the main waveform indicators is quantified in Figure 9.

### 3.3. Speed-Dependent Enhancement of Conductive-Carrier-Induced Waveform Reconstruction

The speed-dependent response was investigated at a fixed carrier geometry. Increasing the scanning speed changes the measured waveforms of both aluminum and copper carriers. At lower speeds, the responses retain a localized peak-valley structure. As speed increases, the main peak becomes more strongly modulated, the post-peak response remains elevated over a longer scanning distance, and downstream recovery becomes delayed. The difference signals relative to the air reference become stronger around the main response and downstream recovery region, especially for copper.

Quantitative indicators further confirm the dynamic enhancement. Relative peak change, peak-position shift, and normalized distortion energy increase with velocity, while the negative-valley contribution is redistributed. Copper produces stronger carrier-induced reconstruction than aluminum because of its higher conductivity. These results indicate that speed does not introduce an independent waveform mode; it amplifies the same conductive-boundary-induced reconstruction mechanism identified in the material comparison.

**Figure 10:**
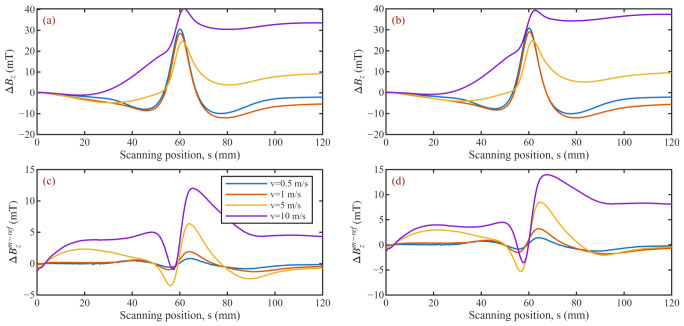
Speed-dependent waveform evolution at *t_s_* = 5 mm. (**a**) Δ*B_z_* waveforms of the aluminum carrier under different velocities. (**b**) Δ*B_z_* waveforms of the copper carrier under different velocities. (**c**) Difference signals between aluminum and air. (**d**) Difference signals between copper and air. The conductive-carrier contribution increases with scanning speed and becomes more evident in the main-peak and downstream recovery regions.

The velocity-dependent waveform evolution for aluminum and copper carriers is shown in Figure 10.

**Figure 11:**
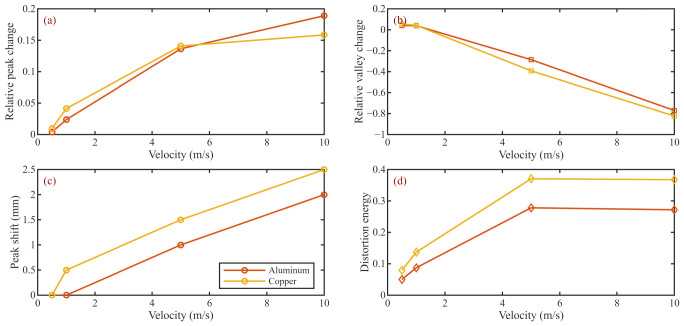
Quantitative indicators of speed-dependent conductive-carrier-induced waveform reconstruction at *t_s_* = 5 mm. (**a**) Relative peak change. (**b**) Relative negative-valley change. (**c**) Peak-position shift. (**d**) Normalized distortion energy. The increasing peak modulation, peak shift, and distortion energy demonstrate the dynamic enhancement of the conductive-carrier-induced waveform reconstruction.

The corresponding speed-dependent quantitative indicators are summarized in Figure 11. Figure 11 should be interpreted as a quantitative result of the present axisymmetric ring-like carrier. In a practical three-dimensional open holder, finite ends, gaps, slots, and interrupted current paths may change the effective loop resistance, conductive-volume participation, current centroid, and sensor-carrier coupling distance. Thus, the exact magnitudes and slopes in Figure 11 are model-specific, whereas the qualitative trend that increasing scanning speed enhances carrier-induced peak modulation, feature-position shift, distortion energy, and downstream recovery remains physically relevant.

### 3.4. Geometry-Dependent Regulation and Simulation Summary

The concave carrier geometry was further evaluated through bottom thickness *h_b_*, bottom width *w_b_*, side-wall height *h_s_*, and side-wall thickness *t_s_*. These dimensions change the effective conducting path, conducting cross-section, local resistance, current centroid, and coupling distance between the induced current and Point A. The results indicate that carrier geometry is an electromagnetic design variable rather than a purely mechanical dimension. Increasing a geometrical dimension does not necessarily strengthen or weaken the waveform reconstruction monotonically because it modifies conductive volume, loop resistance, current-path distribution, and field-coupling distance simultaneously.

The simulation results establish a consistent mechanism chain. Carrier conductivity controls induced-current strength. Scanning speed controls dynamic enhancement and relaxation-related tailing. Carrier geometry controls current-path distribution and coupling distance. These factors jointly determine the final waveform reconstruction.

**Figure 12:**
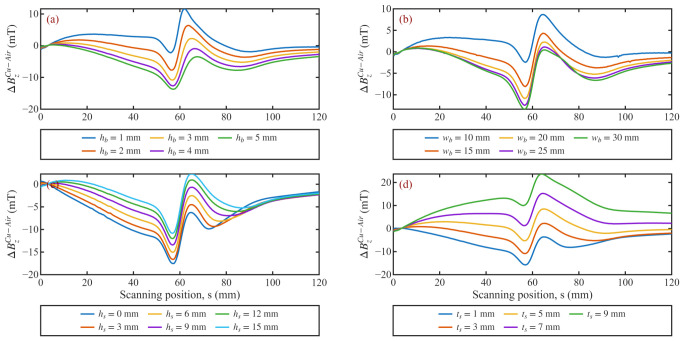
Geometry-dependent regulation of the conductive-carrier-induced waveform reconstruction. (**a**) Influence of bottom thickness *h_b_*. (**b**) Influence of bottom width *w_b_*. (**c**) Influence of side-wall height *h_s_*. (**d**) Influence of side-wall thickness *t_s_*.

The geometry-parametric results in Figure 12 should also be interpreted within the axisymmetric ring-of-revolution representation. In this representation, the concave U-shaped carrier is circumferentially continuous, and the dominant eddy-current paths are therefore constrained to close along idealized ring-like loops. A practical three-dimensional probe holder usually has finite axial and circumferential extents, assembly gaps, slots, screw holes, wiring exits, and interrupted conductive paths, which can modify loop resistance, current-centroid location, conductive-volume participation, and sensor-carrier coupling distance. Therefore, the absolute values of peak modulation, peak-position shift, normalized distortion energy, and the locations or rankings of non-monotonic extrema in the geometry-parametric curves should not be directly transferred to an arbitrary open three-dimensional holder. The qualitative trends expected to remain physically relevant are the dependence of the response on conductive volume, loop resistance, current centroid, and sensor-carrier coupling distance, as well as the concentration of waveform changes around high-gradient and downstream-recovery regions. Quantitative transfer to a specific open carrier requires a full three-dimensional electromagnetic model.

The geometry-dependent regulation caused by the concave carrier dimensions is summarized in Figure 12.

**Figure 13:**
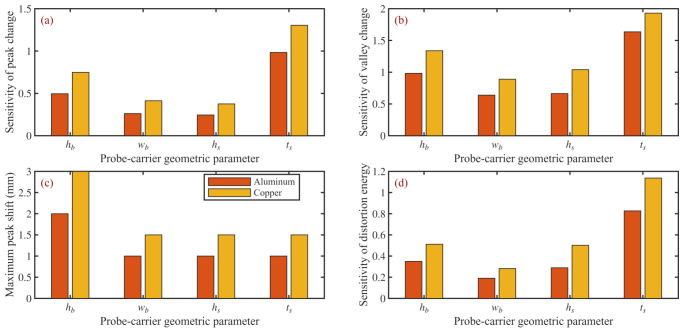
Quantitative sensitivity analysis of waveform indicators with respect to geometric parameters. (**a**) Sensitivity of relative peak change. (**b**) Sensitivity of relative valley change. (**c**) Maximum peak-position shift. (**d**) Sensitivity of normalized distortion energy. The bar charts compare the responses of aluminum and copper carriers under variations of the probe-carrier geometric parameters *h_b_*, *w_b_*, *h_s_*, and *t_s_*.

The overall simulation-based interpretation of material, speed, and geometry effects is summarized in Figure 13.

## 4. Experimental Validation

### 4.1. Experimental Configuration and Validation Route

Experimental validation was conducted on a ferromagnetic rod specimen to examine whether a conductive near-field boundary surrounding the sensing element can reproducibly reconstruct the measured MFL waveform. Two configurations were compared: a reference configuration without an additional conductive boundary adjacent to the sensing region and a conductive-boundary configuration in which the conductive boundary material used in the experiment was placed near the sensing element. The excitation unit, sensing position, lift-off, scanning path, conditioning circuit, acquisition channel, and data-acquisition settings were kept unchanged. The experimental scanning speed and sampling frequency are now explicitly reported as *v_exp_* = 1 m/s and fs = 2000 Hz, respectively, because these two quantities determine both the physical time represented by the sample-domain analysis and the diffusion-scanning ratio discussed in Section 4.4.

For each configuration, a long-distance scanning record containing repeated defect-related responses was acquired. Each cropped signal was mildly baseline-corrected by subtracting the median value of the low-amplitude samples below the 45th percentile of the absolute-amplitude distribution. The subsequent processing route consisted of repeated-event extraction, event resampling, peak-based alignment, mean and standard deviation calculation, amplitude-matched residual analysis, event-level indicator extraction, relaxation-style fitting, and gradient-domain comparison. The principal experimental and processing parameters are summarized in Table 2 to improve reproducibility.

To clarify the relationship between numerical and experimental configurations, it should be noted that the defect geometries used in the simulations and experiments are intentionally different. The finite-element model adopts a local surface defect with a length of 2 mm and a depth of 2 mm, which is used to isolate the carrier-induced waveform reconstruction mechanism under controlled conditions. In contrast, the experimental specimen employs a 5 mm diameter, 2 mm deep artificial defect to ensure stable and repeatable defect-related responses under repeated-event acquisition. Therefore, the comparison between numerical and experimental results is performed at the level of waveform reconstruction mechanisms and indicators, rather than one-to-one geometric equivalence.

**Figure 14:**
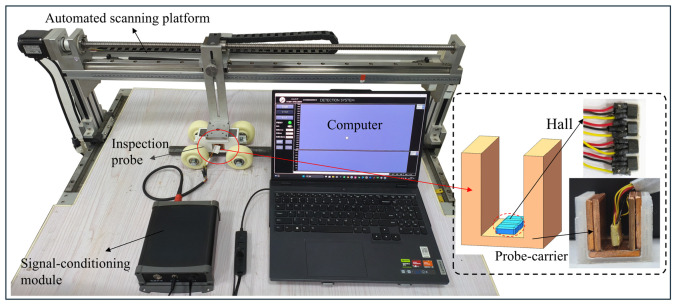
Experimental setup for validating conductive-carrier-induced waveform reconstruction using a simplified ferromagnetic rod specimen. The setup includes the automated scanning platform, inspection probe, signal-conditioning module, computer-based data-acquisition system, and the probe-carrier region with the Hall sensing element. The enlarged inset shows the local arrangement of the Hall sensor and probe carrier used to introduce the conductive near-field boundary near the sensing element.

The experimental platform and validation route are shown in Figure 14.

### 4.2. Raw Response and Repeated-Event Consistency

The cropped reference response contains repeated waveform events with clear positive and negative peaks. After the conductive boundary is introduced, the repeated defect-related responses remain identifiable, confirming that the original magnetic response is retained. However, the waveform morphology is no longer a simple scaled version of the reference response. The affected response exhibits a lower peak-to-peak amplitude, modified local lobes, and a more extended recovery region after the main response.

Repeated-event extraction was performed algorithmically to avoid arbitrary manual selection. Five response events were identified from each record using the same detection, segmentation, resampling, and alignment procedure. These five events should be interpreted as repeated-event consistency evidence obtained under the same hardware, lift-off, scanning path, and acquisition settings. They are not intended to represent population-level statistical sampling. Under this controlled repeated-event setting, the affected events consistently show lower peak levels and broader post-peak regions, supporting the reproducibility of the carrier-induced waveform modification.

**Figure 15:**
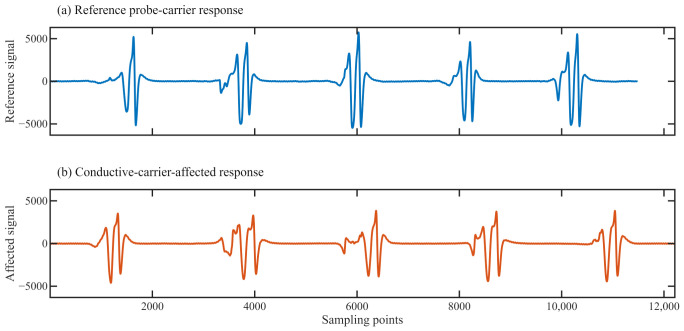
Raw experimental responses under the reference and conductive-carrier-affected conditions. (**a**) Reference probe-carrier response. (**b**) Conductive-carrier-affected response.

The raw responses under the reference and conductive-carrier-affected conditions are compared in Figure 15.

**Figure 16:**
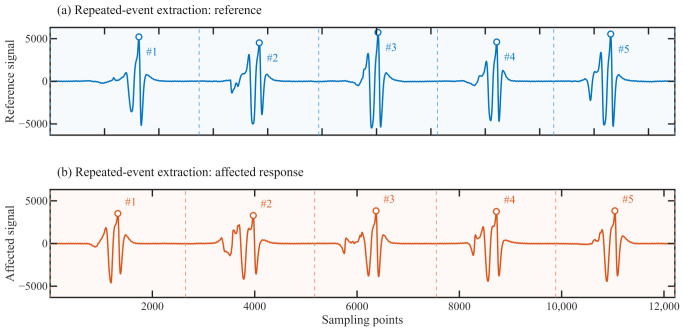
Repeated-event extraction from the experimental scanning records. (**a**) Reference response. (**b**) Conductive-carrier-affected response.

The repeated-event extraction results are shown in Figure 16.

### 4.3. Mean Response, Residual Reconstruction, and Waveform Indicators

The extracted events were resampled to a common length and aligned by the dominant positive peak. Specifically, the common length was set as the rounded median of all extracted event lengths and was constrained to be not less than 1000 samples. Peak alignment was limited to 10% of the common length to avoid excessive phase correction. The reference cycles show consistent main features, while the conductive-carrier-affected cycles preserve the event morphology but show a reduced main response and a wider disturbed region. The averaged waveforms and their standard-deviation envelopes therefore provide a stable representation of the repeated-event carrier-induced modification.

To distinguish waveform reconstruction from uniform attenuation, a scaled residual was calculated as

(31) $$R\left(x\right)=B_{aff}\left(x\right)-\alpha B_{ref}\left(x\right).$$

with

(32) $$\alpha =\frac{\int_{\Omega_{a}}B_{aff}\left(x\right)B_{ref}\left(x\right)dx}{\int_{\Omega_{a}}B_{ref}^{2}\left(x\right)dx}$$

The scale factor alpha removes the best-fit global gain difference between the two mean responses. Therefore, if the conductive boundary only caused uniform attenuation, the residual after amplitude matching would approach zero over the active response region. The residual remains non-zero and is concentrated around the main response and the downstream recovery region, indicating structured waveform reconstruction rather than simple amplitude reduction.

Four indicators were extracted from each aligned event:

(33) $$A_{pp}=B_{max}-B_{min}.$$

(34) $$W_{10}=x_{R,10}-x_{L,10}$$

(35) $$I_{TA}=\frac{x_{R,10}-x_{p}}{x_{p}-x_{L,10}}.$$

Here, *x_L_*_,10_ and *x_R_*_,10_ are the left and right positions where the waveform magnitude decreases to 10% of the peak response level, and *x_p_* is the dominant peak position. The indicators are reported at the repeated-event level and summarized by their mean and standard deviation for the five extracted events. The affected response exhibits reduced *A_pp_*, increased *W*_10_, redistributed zero-crossing position x_0_, and enhanced *I_TA_*. These simultaneous changes support the secondary-field-mediated reconstruction mechanism while making clear that the experimental statistics represent repeated-event consistency under one operating condition.

**Figure 17:**
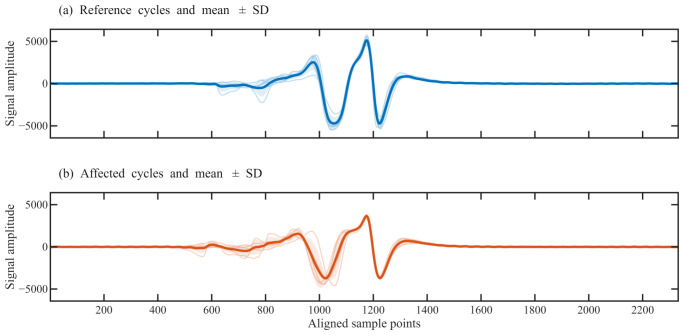
Aligned repeated cycles and mean responses. (**a**) Reference cycles and mean response with standard-deviation envelope. (**b**) Conductive-carrier-affected cycles and mean response with standard-deviation envelope. Five repeated events were used for each condition.

The aligned cycles and mean responses used for statistical comparison are shown in Figure 17.

**Figure 18:**
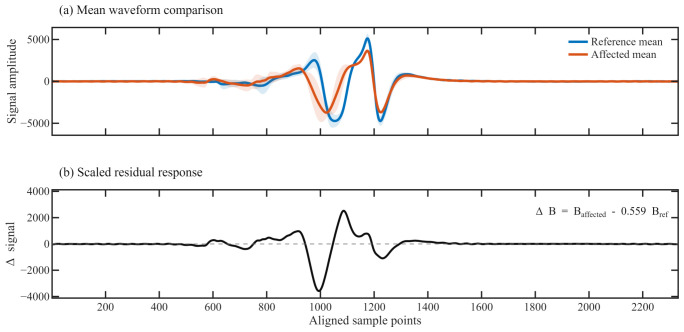
Mean waveform comparison and scaled residual response. (**a**) Comparison between the reference mean response and the conductive-carrier-affected mean response. (**b**) Scaled residual response after subtracting the amplitude-matched reference response. The non-zero structured residual indicates waveform reconstruction beyond uniform attenuation.

The mean-waveform comparison and scaled residual response are given in Figure 18.

**Figure 19:**
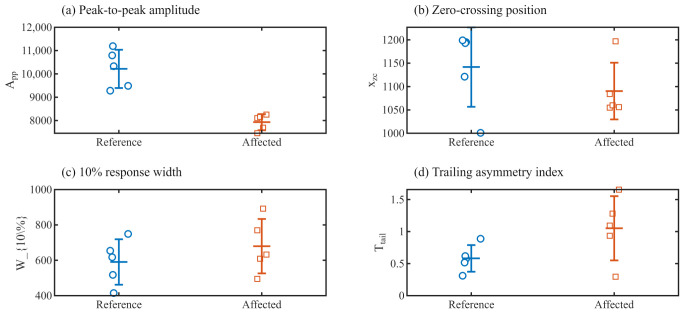
Quantitative comparison of waveform indicators between the reference and conductive-carrier-affected responses. (**a**) Peak-to-peak amplitude. (**b**) Zero-crossing position. (**c**) 10% response width. (**d**) Trailing asymmetry index. The scatter points are the five repeated events, and the error bars denote mean +/− standard deviation.

The event-level waveform indicators are compared in Figure 19.

### 4.4. Experimental Operating Point and Relation to the Diffusion-Scanning Criterion

The speed dependence predicted in the theoretical section is described by the diffusion-scanning ratio $\Lambda (\tau )=v\tau /W_{d}$. In this criterion, *v* is the scanning speed, τ is a generalized memory timescale relevant to the physical process under consideration, and W_d_ is the characteristic spatial width of the defect response. Physically, vτ is the distance travelled by the probe during one memory-relaxation time. Thus, $\Lambda \left(\tau \right)$ indicates whether the carrier-induced eddy-current memory decays within a fraction of the defect-response width or persists over a comparable downstream distance.

For the experimental record, the operating point is evaluated at $v_{exp}$ = 1 m/s, fs = 2000 Hz, and *W_d_* = 702 samples. At this scanning speed and sampling frequency, the spatial resolution is 0.5 mm/sample, yielding a physical characteristic response width of *W_d_* ≈ 0.351 m. To avoid ambiguity in statistical interpretation, *W_d_* is defined as a waveform-level characteristic width extracted from the averaged response. Event-level variations are treated separately in the repeated-event statistical analysis and are not used in the definition of *W_d_*. The present experiment was performed at one representative scanning speed rather than over a velocity series. Consequently, it verifies the existence and repeated-event reproducibility of carrier-induced waveform reconstruction at this operating point, whereas the full transition with increasing $\Lambda \left(\tau \right)$ is supported primarily by the velocity-dependent finite-element results in Section 3.3.

The material-diffusion timescale is estimated using the one-dimensional through-thickness approximation $\tau_{d,est}\approx \mu_{c}\sigma_{c}l_{c}^{2}/\pi^{2}$, where $l_{c}=h_{b}$ is taken as the characteristic conducting thickness of the conductive boundary nearest to the sensing region. This estimate represents a lower-bound diffusion time, describing simplified through-thickness magnetic diffusion only. It does not include contributions from three-dimensional current-loop closure, distributed current paths, sensor-carrier coupling distance, or signal-processing effects.

Using this estimate, the corresponding diffusion-based scanning ratio is $\Lambda_{d,est}=v_{exp}\tau_{d,est}/W_{d}\approx$ 1.89 × 10^−4^. This value indicates that pure through-thickness diffusion alone places the system deeply inside the weak-memory regime. Therefore, $\Lambda_{d,est}$ is reported only as a material-diffusion reference point and should not be interpreted as a quantitative explanation of the experimentally observed waveform reconstruction.

**Figure 20:**
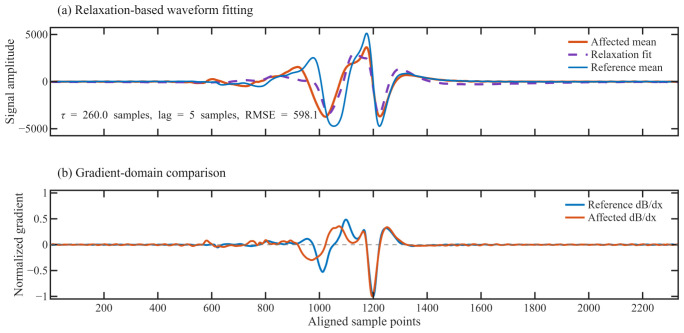
Relaxation-based fitting and gradient-domain comparison. (**a**) Relaxation-style fitting of the conductive-carrier-affected mean response. The fitted time constant and lag are reported in samples and should be converted to physical time using fs. (**b**) Normalized gradient comparison between the reference and affected mean responses.

In contrast, the relaxation-style fitting shown in Figure 20 yields a fitted memory parameter $\tau_{fit}$ = 260/fs = 130 ms. This parameter is interpreted as an effective waveform-memory time of the complete measurement chain ($\tau_{eff}$) rather than a material constant of the conductive boundary. It reflects the combined influence of material diffusion, finite three-dimensional current closure, distributed current-loop paths, sensor-carrier coupling distance, spatial sampling, event alignment, and signal processing effects.

The corresponding effective scanning ratio is $\Lambda_{fit}=v_{exp}\tau_{fit}/W_{d}\approx$ 0.37. Unlike the material-diffusion estimate, this order-unity value is consistent with the experimentally observed waveform broadening, peak shift, and trailing asymmetry. However, since $\Lambda_{fit}$ is obtained from waveform fitting rather than direct physical measurement, it is interpreted as a phenomenological consistency indicator of effective memory, not as an independent validation of the diffusion criterion.

Overall, the two timescales serve distinct roles: $\tau_{d,est}$ defines a lower-bound material diffusion reference, while $\tau_{fit}$ represents the effective memory of the full measurement chain. Accordingly, the diffusion-scanning criterion in this work is formulated as a general framework $\Lambda (\tau )=v\tau /W_{d}$, where the physical interpretation depends on the selected timescale. The present experiment thus confirms carrier-induced waveform reconstruction at a single operating point, while the full regime transition is governed by the velocity-dependent behavior demonstrated in Section 3.3. The experimental operating point, diffusion-scanning ratios, and relaxation-fit quantities used in this interpretation are summarized in Table 3.

### 4.5. Relaxation-Style Fitting and Gradient-Domain Evidence

The relaxation-style fitting and gradient-domain comparison provide further evidence for the induction-based mechanism. The affected mean response can be represented by a finite-memory form derived from the reference response, especially in the delayed recovery and post-peak extension regions. The fitted parameter reported in Figure 20 is $\tau_{fit}$ = 260 samples. To provide a physically interpretable time scale, this value is converted to physical time using the sampling frequency fs, yielding $\tau_{fit,time}$ = 260/fs. The fitted lag of 5 samples is similarly converted as 5/fs.

The RMSE reported in Figure 20 is expressed in the original signal-amplitude unit. To ensure comparability between different experimental conditions, the normalized root mean square error is evaluated as $NRMSE=RMSE/A_{pp,affected}$, where $A_{pp,affected}$ is the peak-to-peak amplitude of the affected mean response. The relaxation fit is interpreted as evidence of effective waveform memory in the measured response. This memory effect encapsulates the combined influence of material diffusion, finite three-dimensional current closure, sensor-carrier coupling distance, spatial sampling, and signal processing. Therefore, the fitted parameter $\tau_{fit}$ represents an effective memory of the complete measurement chain, rather than a pure material diffusion constant of the conductive carrier. The significant difference between $\tau_{fit}$ and the theoretical material-diffusion estimate $\tau_{d,est}$ demonstrates that the simplified one-dimensional through-thickness diffusion model substantially underestimates the macroscopic electromagnetic memory of the 3D system. Accordingly, $\tau_{d,est}$ and $\tau_{fit}$ are utilized in this study to explicitly separate the material-diffusion lower bound from the system-level memory timescale, rather than to serve as a quantitative validation between them.

For a moving conductive carrier, the induced response is closely related to the spatial variation of the magnetic field along the scan path. The main discrepancy between the reference and affected responses appears around the high-gradient region of the principal waveform, and the affected response shows redistributed gradient peaks after normalization. Together with the scaled residual, repeated-event indicators, $\Lambda_{d}$ operating-point check, and relaxation-style fit, these results confirm that the conductive near-field carrier produces structured waveform reconstruction rather than simple amplitude reduction.

The relaxation-based fitting and gradient-domain evidence are shown in Figure 20.

## 5. Discussion

These results clarify the scientific novelty of the present study. The underlying electromagnetic mechanism is based on classical induction and eddy-current secondary-field superposition; the contribution is sensing-system level rather than a new electromagnetic law. In conventional MFL interpretation, the probe carrier is often regarded as a mechanical support, lift-off-maintaining structure, or passive auxiliary component. The present results show that a conductive carrier located close to the sensing element can instead act as an active near-field conductive boundary. Its induced current and secondary magnetic field participate in signal formation, and the resulting effect depends on local field gradients, carrier conductivity, scanning velocity, current-loop geometry, and sensor-carrier coupling distance. Consequently, the measured response is reconstructed through peak modulation, feature-position shift, residual mismatch, response broadening, and trailing-side extension rather than being modified by simple proportional attenuation.

The consistency among the theoretical analysis, finite-element simulations, and experiments is specified here at the mechanism and waveform-indicator levels rather than as one-to-one quantitative equivalence. For the theory–simulation comparison, the reduced model predicts that a moving conductive carrier converts spatial leakage-field gradients into eddy currents and a secondary magnetic field, with finite relaxation producing downstream memory. The finite-element results reproduce this mechanism by showing that the copper and aluminum carriers deviate from the air reference mainly in the high-gradient, main-response, and downstream-recovery regions; the reduced calculation and the finite-element result therefore agree at the mechanism and staged-waveform levels. For the simulation–experiment comparison, the finite-element results show peak modulation, feature-position shift, response broadening, and downstream tailing, while the experiment shows reduced peak-to-peak amplitude, non-zero amplitude-matched residuals, increased response width, redistributed zero-crossing position, and enhanced trailing asymmetry. Thus, the simulation and experiment are consistent at the observable waveform-indicator level, although the experiment validates one representative operating point rather than the full velocity and geometry parameter space. For the theory–experiment comparison, the theoretical predictions of non-proportional waveform reconstruction, residual waveform deviation after amplitude matching, relaxation-related broadening, and trailing-side memory are all observed experimentally.

The findings have direct implications for probe-carrier design. The engineering implication of this system-level novelty is that the probe carrier should be designed as an electromagnetic component rather than only as a mechanical holder. Carrier material should be selected not only according to mechanical strength, wear resistance, and manufacturability but also according to electromagnetic transparency. High-conductivity materials may introduce stronger secondary fields when placed close to the sensor. Low-conductivity materials, insulating inserts, slotted structures, and current-loop-interruption designs can reduce carrier-induced waveform deviation. Carrier geometry should also be optimized as an electromagnetic variable. Increasing a dimension does not necessarily strengthen or weaken the reconstruction strength monotonically; it changes conductive volume, loop resistance, current centroid, and coupling distance simultaneously.

The scanning velocity must be considered together with material and geometry. A probe carrier that performs acceptably under slow laboratory scanning may produce stronger waveform reconstruction under higher industrial scanning speed. The scanning velocity must be considered together with material and geometry. A probe carrier that performs acceptably under slow laboratory scanning may produce stronger waveform reconstruction under higher industrial scanning speed. The diffusion-scanning ratio provides a practical criterion for identifying relaxation-related waveform-memory effects, but its interpretation depends strictly on the selected timescale. The material-diffusion estimate $\Lambda_{d,est}$ represents a lower-bound through-thickness diffusion process of the conductive carrier, whereas the experimentally observed waveform broadening and trailing behavior are more consistently described by the effective waveform-memory ratio $\Lambda_{fit}$, which is evaluated using the fitted system-level memory time $\tau_{fit}$. Therefore, the diffusion-based estimate reflects a material-level limit, while the fitted response captures the effective electromagnetic memory of the complete measurement chain.

The present study uses a simplified rod specimen, one representative experimental scanning speed, five repeated events per condition, and a two-dimensional axisymmetric finite-element model to isolate the carrier-induced mechanism. The five repeated events demonstrate repeated-event consistency under controlled settings, but they should not be interpreted as population-level statistics. More importantly, the axisymmetric concave carrier represents a ring of revolution and therefore imposes an idealized circumferentially closed conducting path. In a practical open three-dimensional probe holder, finite axial ends, assembly gaps, slots, screw holes, wiring exits, and interrupted conductive paths may redistribute the induced current and change the effective loop resistance, conductive-volume participation, current centroid, and sensor-carrier coupling distance. Therefore, the absolute magnitudes, slopes, peak-position shifts, normalized distortion energy, and non-monotonic geometric extrema obtained from the velocity- and geometry-parametric finite-element results should be interpreted as model-specific rather than as direct quantitative predictions for an arbitrary open holder. Nevertheless, the underlying trends—conductivity- and speed-enhanced carrier participation, geometry-controlled current redistribution, downstream recovery associated with finite relaxation, and the dependence on conductive volume, loop resistance, current centroid, and sensor-carrier coupling distance—are expected to remain physically relevant. Quantitative prediction for a specific practical carrier requires a full three-dimensional electromagnetic model and further multi-speed experimental validation.

## 6. Conclusions

This study investigated the eddy-current-induced waveform reconstruction caused by a metallic probe carrier in MFL inspection. The probe carrier should be treated as an active electromagnetic element influencing MFL signal formation. When it moves through a spatially non-uniform leakage field, motion-induced eddy currents are generated inside the carrier, and the resulting secondary magnetic field superposes with the original defect leakage field.

A mechanism-oriented theoretical framework was established using generalized induction, secondary-field superposition, dynamic source evolution, and relaxation-controlled memory. The model shows that the carrier-induced effect is governed by material conductivity, scanning velocity, carrier geometry, and sensor-carrier coupling distance. The generalized diffusion-scanning ratio $\Lambda \left(\tau \right)$ further describes when the carrier memory becomes comparable to the time required for the probe to pass through the defect-response width and therefore when peak shift, response broadening, and downstream tailing are expected to become significant.

Transient finite-element simulations demonstrated that aluminum and copper carriers reconstruct the waveform relative to the Air reference. This reconstruction is reflected by valley modification, main-peak modulation, feature-position shift, and downstream tailing. Higher scanning speed increases peak modulation, peak shift, and distortion energy. Carrier geometry regulates the response by changing the effective current path, conducting cross-section, loop resistance, and coupling distance.

Experimental results further corroborated the mechanism at one representative operating condition. The conductive-carrier-affected response shows reduced peak-to-peak amplitude, non-zero scaled residuals after amplitude matching, broadened response width, shifted feature positions, and enhanced trailing asymmetry. Repeated-event extraction, mean-waveform comparison, relaxation-style fitting, gradient-domain analysis, and the added $\Lambda \left(\tau \right)$ operating-point interpretation consistently show structured waveform reconstruction rather than simple amplitude attenuation. Because the experiment used one scanning speed and five repeated events per condition, the conclusion is limited to mechanism-level corroboration and repeated-event consistency; multi-speed experiments are required to map the full $\Lambda \left(\tau \right)$ regime transition.

The metallic probe carrier should therefore be considered an active electromagnetic participant in near-field MFL measurement. This conclusion provides a basis for low-interference carrier design, electromagnetic material selection, speed-dependent compensation, and more reliable interpretation of magnetic-sensor signals in steel-wire-rope MFL inspection. The study also clarifies that the experimental section demonstrates a representative carrier-induced effect, while broader quantitative generalization requires additional three-dimensional modeling and multi-speed experimental validation. The quantitative indicator values obtained from the axisymmetric carrier should be interpreted as model-specific, whereas the qualitative mechanism trends provide guidance for three-dimensional carrier design and require further validation by full three-dimensional modeling.

## Figures and Tables

**Table 1:** Main parameters and variables used in the transient finite-element simulations.

Category	Parameter	Value or Setting
Model type	Geometry assumption	2D axisymmetric transient finite-element model
Specimen	Material and magnetic property	1020 steel; B-H curve and derived *μ*-H curve shown in Figure 6c
Specimen	Dimensions	Radius 15 mm; length 500 mm
Permanent magnet	Type and residual flux density	N52 permanent magnet; $B_{r}=1.44\;T$
Permanent magnet	Dimensions	Axial length 60 mm; inner radius 35 mm, outer radius 50 mm
Yoke	Material and dimensions	1020 steel; two yokes, each 15 mm axial length and 15 mm radial height
Defect	Type and dimensions	Local surface defect; length 2 mm; depth 2 mm
Probe carrier	Shape	Concave carrier
Probe carrier	Baseline geometry	$h_{b}$ = 3 mm, $w_{b}$ = 20 mm, $h_{s}$ = 15 mm, $t_{s}$ = 3 mm
Probe carrier	Geometry variables	$h_{b}$, $w_{b}$, $h_{s}$, $t_{s}$
Carrier material properties	Reference/conductive cases	air: *σ* = 0 S/m; aluminum: *σ* = 3.774 × 10^7^ S/m; copper: *σ* = 5.998 × 10^7^ S/m; *μ_r_* ≈ 1.
Motion	Velocity	v = 0.5, 1, 5, and 10 m/s
Extraction point	Point A	Located in the carrier recess; 0.5 mm above the carrier bottom
Output	Field and processing	$B_{z}$ at Point A
Numerical implementation	Software, physics, boundary, and mesh	COMSOL Multiphysics 6.2; 2D axisymmetric magnetic fields model with moving mesh; large air domain; default magnetic-insulation outer boundary; local refinement near defect, carrier, and Point A, minimum element size approximately 0.25 mm.
Numerical implementation	Study sequence, motion, and time	Stationary solution used as initial condition for the time-dependent step; axial moving-mesh velocity *v* was applied to the steel-rod/defect region; total travel *s* = *vt* = 120 mm, i.e., T = 0.12/*v*; the output time range was defined as 0: T/240: T.

**Table 2:** Experimental configuration and data-processing parameters used for validating conductive-carrier-induced waveform reconstruction.

Parameter or Process Item	Value or Setting
Specimen and defect source	Specimen material: 1020 steel; specimen diameter: 15 mm; specimen length: 500 mm; artificial defect diameter: 5 mm; defect depth: 2 mm.
Excitation and sensing configuration	Permanent magnet: N52; yoke material: 1020 steel. Both the permanent magnet and yoke had an inner diameter of 70 mm and an outer diameter of 100 mm. The Hall sensor was placed at the center of the conductive-boundary configuration, 0.5 mm above the inner bottom surface of the conductive boundary.
Conductive-boundary configuration	Copper conductor; bottom width (*w_b_* = 25) mm, bottom thickness (*h_b_* = 3) mm, side-wall height (*h_s_* = 25) mm, and side-wall thickness (*t_s_* = 6) mm.
Experimental scanning speed	*v_exp_* = 1 m/s
Sampling frequency	fs = 2000 Hz
Baseline correction	Median subtraction using low-amplitude samples below the 45th percentile of ∣B∣.
Repeated-event extraction	Five repeated events per condition; peaks were detected after seven-point moving-average smoothing with a 35% peak-height threshold; adjacent events were separated by midpoints between detected peaks.
Event resampling and alignment	Events were resampled to a common length and aligned by the dominant positive peak; the maximum allowed shift was 0.10 *N_norm_*.
Waveform comparison and indicators	The affected mean waveform was compared with the least-squares amplitude-scaled reference mean waveform; indicators included *A_pp_*, *x*_0_, *W*_10_, and *I_T__A_*.
Relaxation-style fitting	Finite-memory fitting was performed over *τ_fit_* = 5–260 samples and lag = −120–120 samples; fitted parameters were converted to physical time using the sampling rate.

**Table 3:** Experimental operating point, diffusion-scanning ratio, and relaxation-fit quantities.

Quantity	Value
Experimental scanning speed	*v_exp_* = 1 m/s
Sampling frequency	fs = 2000 Hz, corresponding to 0.5 ms per sample and 0.5 mm per sample.
Characteristic response width	$W_{d}=W_{10,affected\;mean}=702\;samples,$ corresponding to 0.351 m; measured from the averaged affected waveform
Theoretical diffusion time	$\tau_{d,est}\approx \mu_{c}\sigma_{c}l_{c}^{2}/\pi^{2}=0.0665\;ms,$ assuming copper with $\sigma_{c}=5.8\times 10^{7}\;S/m,$ $\mu_{c}=\mu_{0},$ and $l_{c}=h_{b}=3\;mm.$
Fitted effective waveform-memory time	$\tau_{fit}$ = 260/fs = 130 ms; interpreted as an effective measurement-chain memory, not a copper material constant
Experimental diffusion-scanning ratio	$\Lambda_{d,exp}=v_{exp}\tau_{d,est}/W_{d}=1.89\times 10^{-4};$ used only to locate the pure material-diffusion operating point
Effective waveform-memory ratio	$\Lambda_{fit}=v_{exp}\tau_{fit}/W_{d}\approx 0.37$
Fitted lag	lag = 5/fs = 2.5 ms
RMSE and normalized RMSE	RMSE = 598.1 in the original signal-amplitude unit; NRMSE=598.1/7385.51=0.0810=8.10.

## Data Availability

The data presented in this study are available from the corresponding author upon reasonable request.

## Abbreviations

The following abbreviations and nomenclature are used in this manuscript:

$\Omega_{c}$	Conductive domain of the metallic probe carrier
$\sigma_{c}$	Electrical conductivity of the carrier
$\mu_{c}$	Magnetic permeability of the carrier
v	Scanning velocity of the carrier relative to the specimen
*v_cr_*	Critical scanning velocity associated with the diffusion-scanning transition criterion
$J_{c}$	Induced current density in the carrier
φ_c_	Electric scalar potential in the carrier
$A_{s}$	Magnetic vector potential associated with the source leakage field
$B_{s}$	Original leakage magnetic flux density without carrier-induced disturbance
$B_{sec}$	Carrier-induced secondary magnetic flux density
$B_{m}$	Magnetic flux density measured at the sensing point
*E_eff_*	Effective non-conservative induction field driving carrier eddy currents
$E_{m}$	Motional electromotive force induced by carrier motion in a non-uniform leakage field
$r_{s}$	Position of the magnetic sensing element
$l_{c}$	Characteristic conducting thickness of the carrier
*L_e_*	Effective length of the dominant eddy-current loop contributing to the sensing-point response
*ρ*_Δ_	Normalized correlation coefficient between waveform deviation signals
*E_mis_*	Normalized waveform mismatch metric between reference and reconstructed responses
*e_p_*	Peak-position shift error
τ	Magnetic diffusion or relaxation timescale of the carrier
*τ_fit_*	Fitted effective waveform-memory time obtained from relaxation-style waveform analysis
*τ_d,est_*	Estimated material magnetic-diffusion time based on one-dimensional through-thickness approximation
$W_{d}$	Dominant spatial width of the defect response
$\Lambda \left(\tau \right)$	Diffusion-scanning ratio
$B_{z}$	Axial magnetic flux density component extracted at Point A
$\Delta B_{z}$	Baseline-subtracted defect-related response
$A_{pp}$	Peak-to-peak amplitude of an extracted event
$x_{0}$	Zero-crossing position
$W_{10}$	10% response width
$I_{TA}$	Trailing asymmetry index
*v_exp_*	Experimental scanning velocity
fs	Sampling frequency of the acquisition system
NRMSE	Normalized root mean square error used for waveform deviation evaluation
